# A large electroencephalographic motor imagery dataset for electroencephalographic brain computer interfaces

**DOI:** 10.1038/sdata.2018.211

**Published:** 2018-10-16

**Authors:** Murat Kaya, Mustafa Kemal Binli, Erkan Ozbay, Hilmi Yanar, Yuriy Mishchenko

**Affiliations:** 1 Mersin University, Mersin, 33140, Turkey; 2 Izmir University of Economics, Izmir, 35330, Turkey

**Keywords:** Computational neuroscience, Biomedical engineering

## Abstract

Recent advancements in brain computer interfaces (BCI) have demonstrated control of robotic systems by mental processes alone. Together with invasive BCI, electroencephalographic (EEG) BCI represent an important direction in the development of BCI systems. In the context of EEG BCI, the processing of EEG data is the key challenge. Unfortunately, advances in that direction have been complicated by a lack of large and uniform datasets that could be used to design and evaluate different data processing approaches. In this work, we release a large set of EEG BCI data collected during the development of a slow cortical potentials-based EEG BCI. The dataset contains 60 h of EEG recordings, 13 participants, 75 recording sessions, 201 individual EEG BCI interaction session-segments, and over 60 000 examples of motor imageries in 4 interaction paradigms. The current dataset presents one of the largest EEG BCI datasets publically available to date.

## Background & Summary

Patients immobilized due to trauma or other medical conditions suffer from a significant deficit of motor and communication functions. Recent advances in neural prosthetics may offer to improve the condition of such patients by allowing them to regain control of certain motor and communication abilities^1–3^. Targeted muscle re-innervations^4^ and myoelectric control based on residual muscle activity^5–7^ offer exciting possibilities for neural prosthetics. Another direction is offered by brain-computer interfaces (BCI) that aim to translate neural activity in the brain into control signals for external devices^8–13^. Important advances in BCI include neural control of robotic actuators in monkeys^14–17^, nonhuman primates^18–20^, as well as humans^21–25^. Recent studies demonstrated high-performance 3D reaching control by tetraplegic individuals using an intracranial BCI^26,27^. In that respect, invasive BCIs present considerable potential for high degree of freedom control of assistive robotic devices^19,26,27^.

Research into BCIs that do not necessitate risky brain surgery is also of great importance, and electroencephalographic (EEG) BCI presents a particularly interesting direction. The key advantages of EEG for BCI are the maturity of the technology, relative ease of use and low costs, as well as the robustness, portability and versatility of recent EEG devices.

Significant progress in EEG BCI has been reported in the literature. The pioneering works of Wolpaw and McFarland have demonstrated 1D, 2D and 3D computer cursor control by using sensorimotor rhythm modulation^25,28,29^. EEG BCIs have been adopted in a number of settings since then. The RIKEN BSI-TOYOTA Collaboration Center has presented an EEG BCI technology for real-time control of motorized wheelchairs. Pfurtscheller *et al.* describe an EEG BCI technique for neurostimulation of a tetraplegic patient’s hand, allowing the patient to grasp objects with that hand^30^. Chae *et al.* have described EEG BCI control of a humanoid robot^31^, and Sankai report progress on the development of an exoskeleton suit with EEG BCI control^32^. Many other exciting developments have been presented in the field^33–38^.

Development of more effective data processing and analysis methods for EEG BCI has been hindered by a lack of large, uniform and accessible datasets. Some EEG BCI datasets are available on the Internet, but most are limited by short recording times, a small number of participants or a small number of BCI signals. For example, the BCI Competition IV dataset is one of the most widely used resources in the EEG BCI data processing literature. This resource contains 3 EEG BCI datasets of which two are for synchronous and one for asynchronous BCI. 9 subjects in total are included with approximately 1 h of EEG BCI recordings and 576 imagery trials per subject, either in 2 (left-right hand motor imagery (MI)) or 4 (variable MI) state BCI interaction paradigms. Other EEG BCI datasets, for example those that can be found at http://www.brainsignals.de or http://www.bnci-horizon-2020.eu, share similar restrictions. Cho *et al.*^39^ describe the largest EEG BCI dataset publically released today. It includes data from 52 subjects, but only 36 min and 240 samples of EEG imagery per subject, and with only a left-right hand MI interaction paradigm. The absence of comprehensive public EEG BCI datasets is a significant drawback for the development of new data analysis methods for EEG BCI.

In this work, we publish an EEG BCI dataset (Data Citation 1) collected during the development of a slow cortical potentials MI EEG BCI in Mishchenko *et al.*^40^. The dataset contains 60 hours of EEG BCI recordings across 75 recording sessions of 13 participants, 60,000 mental imageries, and 4 BCI interaction paradigms, with multiple recording sessions and paradigms of the same individuals. BCI interactions involving up to 6 mental imagery states are considered. On average, 4.8 h of EEG recordings and 4600 mental imagery samples are available per participant. The dataset is one of the largest EEG BCI datasets published to date and presents a significant step from existing datasets in terms of uniformity, longitudinal and lateral coverage, and interaction complexity.

## Methods

### Participants and experimental procedures

All experiments were approved by the Ethics Committees of Toros University and Mersin University in the city of Mersin, Turkey. 13 individuals between the ages of 20 and 35 participated in the study. The participants were all healthy volunteers from students studying in the engineering and science programs of Toros University and Mersin University. The participants included 8 males (61.5%) and 5 females (38.5%). The participants had been screened for the absence of psychiatric conditions, any taken medications, and contraindications to EEG. All participants were informed about the purpose and the procedures of the experiments and had given written consent to the collection of data. The names of all participants have been hereby anonymized. The participants are identified only by their aliases “Subject A” through “Subject M”.

**Figure 1:**
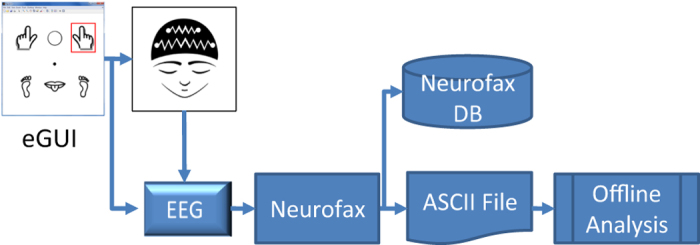
Schematic representation of the data acquisition and processing procedures. First, action signals were presented to participants indicating one of the mental imageries to be implemented. The imagery was implemented by participants once during the period that that action signal remained on. The EEG signal corresponding to the implemented imagery was recorded by EEG-1200 hardware and saved via Neurofax recording software. After the experiment, the acquired EEG data were saved and exported as an ASCII file for further processing. The ASCII data file was imported to Matlab for analysis.

**Figure 2:**
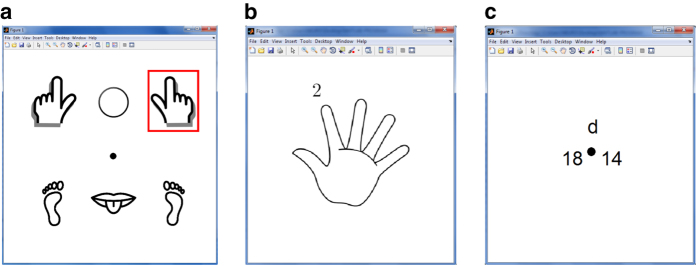
The graphical user interfaces (eGUI) used for BCI interactions. (**a**) For CLA and HaLT interaction paradigms, the eGUI displayed six icons symbolizing left hand, right hand, left leg, right leg, and tongue motor imageries together with a passive imagery indicated by a circle. A fixation point was shown in the center of the screen. Action signals selecting the imagery to be implemented were shown as a red rectangle around the respective motor imagery icon. (**b**) For the 5 F interaction paradigm, the eGUI displayed a hand icon with five fingers. The action signal was shown as a number from 1 to 5 directly above the finger for which motor imagery was to be implemented. (**c**) For the FreeForm interaction paradigm, the eGUI showed a fixation point in the center of the screen, total left and right key-press counts, and the letter of the last key press. The participants pressed keys using their right and left hands voluntarily, in a self-paced manner, while the eGUI kept track of the pressed keys and the key press number.

The general experiment workflow is illustrated in Fig. 1. For all recording sessions, the participants were comfortably seated in a recliner chair with the EEG cap placed on their head and a computer screen – the computer system part of the EEG-1200 EEG system – positioned approximately 200 cm in front at slightly above the eye level. The computer screen showed one of the experiment graphical user interfaces (eGUI) such as shown in Fig. 2. Each eGUI was implemented in Matlab in the format of a window with a set of icons signifying different mental imageries to be executed by the participants and a gaze-fixation point in the center of the window. The participants remained motionless and kept their gaze at the fixation point for the duration of the BCI recording sessions.

Each recording session was organized as a sequence of BCI interaction segments separated by 2 min breaks. At the beginning of each recording session, a 2.5 min initial relaxation period was administered so that the participants could relax and acclimatize to the recording session’s conditions. After the relaxation period, three separate 15 min BCI interaction segments were administered using one of the paradigms described in the section “BCI interaction paradigms.” During the interaction segments, the participants executed approximately 300 trials invoking different mental imageries as instructed by eGUI. Each trial began with the presentation of a stimulus action-signal on the eGUI, indicated by a red rectangle to select one of the icons corresponding to the mental imagery to be implemented. The action signal remained on screen for 1 s, during which time the participants implemented the indicated mental imagery once. A pause of variable duration of 1.5-2.5 s followed, concluding the trial. On average, each trial took 3 s with 300 trials performed within single 15 min interaction segments. Each trial invoked a mental imagery selected uniformly at random according to a pseudorandom number generator. Together with all segments, each recording session lasted between 50 and 55 min and the EEG signals were recorded continuously throughout.

### Data acquisition

EEG data were acquired using an EEG-1200 JE-921A EEG system (Nihon Kohden, Japan). EEG-1200 is a standard medical EEG station used in many hospitals. It provides high-precision EEG signal measurements with up to 38 input channels with the common mode suppression of at least 100 dB, according to EEG-1200’s technical manual. In this work, 19 EEG input leads in the standard 10/20 international system have been used.

The EEG data acquisition was performed with the help of a standard 10/20 EEG cap (Electro-Cap International, USA) with 19 bridge electrodes in the 10/20 international configuration. Before each recording session, participants had their head prepared by an EEG technician who cleaned the head skin surface with a cleansing solution and combed the hair around the locations of the electrodes. Subsequently, the EEG cap was replaced on the participant’s head. The distances between nasion, inion, preauricular points and the cap’s Cz electrode were measured using a measuring tape to ensure the correct positioning of the EEG cap to within ±0.25 cm. Once the EEG cap was placed, the bridge electrodes were filled with conductive electro gel (Elector-Cap International, USA) and the electrode impedance monitored using the impedance-check mode of the EEG-1200 system. After achieving impedances ≤10 kΩ with the impedance imbalance ≤5 kΩ, the preparation was considered complete. The preparations took about 30 min per recording session. Electrode impedance was checked using the EEG-1200’s impedance-check mode again at the end of the recording session, to ensure that good electrical contact remained throughout the recording session’s entire duration.

After the preparations, EEG data were recorded. No electromagnetic shielding or artifact control was attempted for the recordings. The rationale being that the EEG BCI data processing sub-system was expected to cope with this sort of data pollution. EEG data were recorded using the Neurofax recording software shipped with the EEG-1200 system. A modified 10/20 montage was used for the data recording. The modified montage consisted of 19 standard 10/20 EEG leads, two ground leads labeled A1 and A2 (placed at the earbuds), and one bipolar lead X3 used for data synchronization, for a total of 22 input channels recorded. The EEG signal was recorded at sampling rate of 200 Hz unless otherwise indicated in the data file. The reference point for all recordings was “System 0 V” as defined by the EEG-1200’s technical manual at 0.55*(C3 + C4) V.

No custom filtering was applied to the recorded EEG signal. A band-pass filter of 0.53-70 Hz was present in all EEG data recorded at 200 Hz sampling rate in the Neurofax software. A 0.53–100 Hz band-pass filter (the widest choice possible in Neurofax software) was applied to the EEG recordings acquired at 1000 Hz sampling rate. These are the hardware filters and therefore part of all the published records. Additionally, a 50 Hz notch filter is present in the EEG-1200 hardware to reduce electrical grid interference.

The mental imagery program in the recording sessions was controlled by Matlab-based eGUI software. This software recorded the interface screen to enable the sequence of events in each recording session to be reconstructed afterward. We observed a significant difference between the EEG-1200’s signal acquisition box and the main computer clocks, which required the EEG data acquired through the acquisition box and the interaction program recording in Matlab to be synchronized. Specifically, the two clocks differed in time offset as well as speed sufficient to disrupt alignment of stimuli onset times (recorded by Matlab on the main computer) and EEG samples, rendering trials unsuitable for BCI analysis. Therefore, additional hardware was deployed to correct for the difference. The hardware forwarded 1 μV synchronization signals from the main computer’s USB port to the X3 bipolar input on the JE-921A acquisition box. Upon each new presentation of a visual action signal, eGUI forwarded a 500 ms 1 μV pulse signal via the computer’s USB port and Arduino Uno microcontroller to the EEG-1200 X3 bipolar input. The signal was subsequently recorded by Neurofax in the 22^nd^ input channel, appearing in the EEG data record as a series of on/off spikes separated by 500 ms, at the beginning and end of each action signal. This signal was perfectly synchronized with the EEG data and used to align the EEG data samples and the interaction record data maintained by Matlab eGUI.

The EEG data acquired in each recording session were saved to the Neurofax internal database and also exported as an ASCII file. The recording session’s eGUI interaction record was saved to a Matlab data file. The exported ASCII EEG data comprised a table of text listing all sampled EEG voltage values for each time point. The voltage resolution in the exported data was 0.01 μV at 24 bits and the sampling rate was that of the original EEG recording. This information was also indicated in the header of the exported ASCII file. The exported ASCII data were imported to Matlab with the help of a custom script based on *convert_nkascii2mat.m* by Timothy Ellmore available from Beauchamp at OpenWetWare.org. The script read and parsed the original Neurofax ASCII data table, imported the eGUI recording session interaction record, synchronized the two, and output a final file of the complete recording session’s data record.

All data included in the study have passed the pre-specified quality checks including ensuring that no terminal motion and E&M-related interference occurred during data collection, all electrode impedances were below specified levels at the start and end of the recording session, and no other technical issues affecting collected data quality were present. No post-hoc data cuts, for example such as based on quality of separation of mental imageries in recordings post-hoc, were applied to the recordings included in this dataset to present researchers with the least-altered raw data possible.

### BCI interaction paradigms

All data in this dataset were recorded in a synchronous BCI paradigm. Each experimental BCI segment consisted of a series of BCI interaction trials in which a visual action signal was shown on the computer screen instructing the participants to implement a given mental image. The action signal remained on the screen for 1 s during which time the participant implemented the corresponding mental imagery once. After 1 s, the action stimulus disappeared and a random duration off-time of 1.5–2.5 s followed, concluding the trial. The trial procedure was repeated 300 times per interaction segment, resulting in a total segment duration of 15 min. Each recording session contained three interaction segments, for a total BCI interaction time of 45 min per recording session. Due to participant fatigue, the interaction segments were capped at 15 min, and a 2-min rest period administered between interaction segments. During the rest period, participants were allowed to talk and move in their chair. EEG data were continuously acquired during the rest periods. EEG electrode and head skin contact was not compromised during rest periods as was verified by the impedance checks, as described in the “Data acquisition” section.

### Paradigm #1 (CLA)

*General Description*: The motor imagery (MI) interaction paradigm has become popular due to its reliance on voluntary actions, which can be visualized by subjects via the neural mirror system and monitored by EEG via the electrodes placed above the motor areas. The movement of right and left hands, legs and tongue have been commonly employed as a paradigm for BCI interactions in the past. Hand movement is especially potent, due to easily distinguishable activity in the contra-lateral cortical regions responsible for the movement of the limbs, located directly under C3, C4, T3, T4 and Cz sites of the standard 10/20 international system. Thus, EEG-based discrimination of left- and right-hand MI based on contra-laterally localized activity observed via C3 and C4 electrodes has become one of the most easily deployable EEG BCI communication paradigms. Thus, our “Paradigm #1 – CLA (Classical)” includes a similar EEG BCI interaction model based on three imageries of left and right-hand movements and one passive mental imagery in which participants remained neutral and engaged in no motor imagery.

#### Stimuli and Experimental Design

Participants viewed a fixation point in the center of eGUI screen (shown in Fig. 2a). At the beginning of each trial, an action signal indicating left hand, right hand or circle (for passive response) was shown for 1 s. During the time that the action signal remained on, the participants implemented the selected motor imagery once. The left- and right-hand motor imageries were implemented by imagining closing and opening the respective fist once. After implementing the imagery, the participant remained passive until the next action signal was presented. For passive imagery, the participants remained passive and did not engage in any voluntary mental imagery until the beginning of the next trial. A response related to the processing of the “passive” instruction might be still observed in the EEG data.

### Paradigm #2 (HaLT)

#### General Description

This BCI interaction paradigm is an extension of the 3-state CLA paradigm above, where a greater number of imageries was used, including the imagery of left and right leg movement and tongue movement, for a total of six mental states to be used for interaction with BCI.

#### Stimuli and Experimental Design

Participants viewed a central fixation point (shown in Fig. 2a). At the beginning of each trial, an action signal indicating left hand, right hand, left leg, right leg, tongue or a circle (indicating passive response) was presented for 1 s. During the time that the action signal remained on, the participants implemented the selected motor imagery once. The left- and right-hand imageries were invoked as in Paradigm #1. The left and right leg motor imageries were invoked as a brief movement of the leg or foot. The tongue imagery was invoked by imagining a distinct letter or sound being pronounced, such as “el.” For passive imagery, the participants remained passive and did not do anything. One imagery was implemented per action signal presentation. After executing the imagery, participants remained passive until the next action signal presentation.

### Paradigm #3 (5F)

#### General Description

This BCI paradigm was intended to study the possibility of discriminating finer movement imageries via EEG signals, such as the movements of the fingers on one hand. These experiments were acquired at either 200 Hz or 1000 Hz sampling rate, for the purpose of inspecting higher frequency EEG signals.

#### Stimuli and Experimental Design

Participants viewed the 5F-interaction eGUI screen shown in Fig. 2b. The participants were asked to focus on the center of the image for the duration of the recording session. At the beginning of each trial, an action signal appeared (represented by a number from 1 to 5) directly above the finger whose movement imagery was to be implemented. The action signal remained on for 1 s, during which time the participants implemented the corresponding imagery once. The imageries were invoked as a flexion of the corresponding fingers up or down, per the preference of the participant. There was no passive state in this paradigm – each action signal required a response. Single imagery was implemented per action signal. After executing the imagery, participants remained passive until the next action signal presentation.

### Paradigm #4 (FreeForm)

**General Description:** This BCI interaction paradigm was intended to examine discrimination of voluntary motor movements before their physical manifestation, by means of the EEG signals. This line of research was not pursued extensively in this work, and a very small number of recording sessions are available for this paradigm.

#### Stimuli and Experimental Design

Participants viewed the FreeForm-interaction eGUI screen shown in Fig. 2c. The participants were asked to focus their gaze on the fixation point while their hands rested calmly on the computer keyboard. Participants were asked to press “d” or “l” keys voluntarily, by using either their left or right hand, at arbitrary times. The time of the key presses was recorded and used to establish the reference point for the analysis of the EEG key-press waveforms. eGUI kept track of the total number of left and right key presses as well as the identity of the last key, which was displayed on the screen. The participants were asked to perform a similar number of left and right key presses, on average. The neural activity changes due to motor planning and execution could be observed in the EEG data in the times immediately preceding the key presses. This paradigm was self-paced.

### Paradigm #5 (NoMT)

#### General Description

The experiments in this paradigm were performed with the participants prior to beginning the experimental program, as a consistency check and baseline determination for subsequent BCI interaction experiments. These recording sessions were similar to HaLT except that participants were not asked to react to any visual signals appearing on the eGUI screen and thus passively watched the computer screen for the duration of the recording session.

#### Stimuli and Experimental Design

Participants viewed a fixation point in the center of the eGUI screen such as shown in Fig. 2a. At the beginning of each trial, an action signal indicating either the left hand, right hand, left leg, right leg, tongue, or a circle indicating passive response was presented for 1 s. The participants remained passive throughout.

### Code availability

The custom Matlab script nkimport.m was used to process the raw ASCII data obtained from the EEG-1200 device. This script is shared with the dataset. The eGUI programs used in the recording sessions are also included with the dataset. For all other software pieces, including those related to data acquisition or processing, the reader is encouraged to contact the corresponding author directly.

## Data Records

### Distribution for use

The data files for the large electroencephalographic motor imagery dataset for EEG BCI can be accessed via the Figshare data deposition service (Data Citation 1).

### EEG data organization for hand, leg, tongue, and finger movements

The dataset in (Data Citation 1) consists of 75 data files each containing the complete data record of one BCI recording session plus one text description file. Each recording session contains approximately 55 min of recorded EEG data for 3 BCI interaction segments, for the total duration of BCI imagery data of approximately 45 min. Each interaction segment consists of presentations of approximately 300 mental imagery symbols identified by the recording session interaction record (also found in the file). Each recording session is performed for one participant and uses one of the BCI interaction paradigms in the Methods section, identified via a systematic naming convention in the data file name.

All data files are shared in .mat format and contain MATLAB-readable records of the raw EEG data and the recording session’s interaction record. The data in each file are represented as an instance of a Matlab structure named “o,” having the following key fields “id,” “nS,” “sampFreq,” “marker” and “data” (detailed in Table 1).

The recording session information for each data file is indicated in the naming system of the file. The file naming system is designed to allow easy searching and filtering, aimed at batch processing and analysis of the data. The file names are initially grouped by recording session paradigm: 5 F, CLA, HaLT, FreeForm, and NoMT, as described in the Methods section. Subsequently, the name of each file identifies the participant engaged in the recording session, labeled as SubjectA through SubjectM. The calendar date of the recording session is indicated next, as well as the total number of BCI imagery states present in the 0St-6St (0 states-6 states) format. At the end of each file name, a brief mnemonic detail is provided. See Table 2 for detailed explanation of the abbreviations (5 F, CLA, HaLT, etc.) and the mnemonics used in the naming of the files. Table 3 lists the gender and demographic information of the recording sessions’ participants. Table 4 lists all the recording sessions grouped by their BCI interaction paradigm.

Examples of the data file naming:

- 5F-SubjectA-160405-5St-SGLHand-HFREQ.mat – Recording session of subject A that took place on April 5^th^, 2016. Recording session paradigm is 5 F – 5 finger motions on one hand. Five mental imagery types are present (5St = 5 finger movements). All motor imageries are of a single hand (SGLHand). EEG data were acquired at 1000 Hz sampling rate (HFreq).
- CLA-SubjectE-160108-3St-LRHand.mat – Recording session of subject E that took place on January 8^th^, 2016. Recording session paradigm is CLA – discrimination of left- and right-hand movements plus a passive state. Three mental imagery states are present (3St). The recording session mnemonic is Left-Right Hand (LRHand).
- FREEFORM-SubjectC-151210-5St-LRHand.mat – Recording session of subject C that took place on December 10^th^, 2015. Recording session paradigm is FreeForm – detection of voluntary left- and right-hand movements. Two mental imagery states are present (2St). The recording session mnemonic is Left-Right Hand (LRHand).
- HaLT-SubjectJ-161121-6St-LRHandLegTongue.mat – Recording session of subject J that took place on November 21^st^, 2016. Recording session paradigm is HaLT – discrimination of left and right hand, left and right leg, tongue movement, and one passive state. Six mental imagery states are present (6St). The recording session mnemonic is Left-Right Hand, Leg, Tongue (LRHandLegTongue).
- NoMT-SubjectH-160628-0St-NoMotor.mat – Recording session of subject H that took place on June 28^th^, 2016. Recording session paradigm is NoMT – visual stimulus only and no motor actions. No mental imagery states are present (0St). The recording session mnemonic is No Motor.

The fields of the record “o” comprising the data record of each file are as follows. The main fields are “marker” and “data,” which contain the recording session’s eGUI interaction record and the EEG data, respectively. The fields “nS” and “sampFreq” contain the total number of EEG signal samples and the sampling rate expressed in Hz or samples per second. Typically, all EEG data were acquired at a sampling frequency of 200 Hz, unless otherwise indicated in the name of the data file by identifier HFREQ, which indicates a 1000 Hz sampling rate setting. The latter change in the sampling rate is also reflected in the “sampFreq” field of the data record itself.

The “data” field contains the recording session’s EEG data in the format of 2D Matlab array of size nSx22, where each column is the time-series of voltage measurements from a single EEG input lead such as F3 or C3. The ordering of the EEG leads is described in the attached description.txt file and is the same for the entire dataset. The “data” array describes the measured voltage time-series from 19 EEG leads in 10/20 configuration, two ground leads A1-A2, and one synchronization channel X3, as detailed previously in this document. All reported voltage measurements are recorded in microvolts. The 22^nd^ column is the synchronization data from X3 bipolar input port and contains spikes associated with the beginning and end of each action signal presentation period. The 22^nd^ channel does not contain actual EEG data and should not be used for any purposes other than verifying synchronization between “data” and “marker” data, below.

The “marker” field contains the recording sessions’ interaction record. This record is in the form of 1D Matlab array of size nSx1, which contains integer values from 0 to 99. Each value encodes the state of the eGUI at the time mapping to the corresponding EEG data sample in the “data” array at the same time-index location. The marker codes from 1 to 6 encode the visual stimuli directing the participants to implement given mental imageries in the order 1: “left hand,” 2: “right hand,” 3: “passive or neutral,” 4: “left leg,” 5: “tongue,” and 6:“right leg” for CLA, HaLT, and FreeForm recording sessions. For 5 F recording sessions, the codes are 1: “thumb,” 2: “index finger,” 3: “middle finger,” 4: “ring finger,” and 5: “pinkie finger”. Codes greater than 10 indicate service periods including 99: “initial relaxation,” 91: “inter-session breaks,” 92: “experiment end.” Code “0” means “blank” or nothing is displayed in eGUI. The interaction record codes are summarized in Table 5.

The recording sessions in the dataset with the “Inter” identifier were collected using custom software that we call interactive interface, and is described in greater details in Mishchenko *et al.*^40^. The key difference is that Matlab software directly interfaced with Neurofax software to accesses the EEG data in real time to enable real-time feedback to be given in a BCI system. Due to the specifics of real-time interfacing with Neurofax software, that data has a different voltage resolution (0.133 μV vs. 0.01 μV) and dynamic range ( ±121 μV vs. 2 mV) relative to the other data records. In all other respects, however, the data format is identical to the rest of the dataset.

For the analysis of the included data, the times when the “marker” value switched from 0 to a non-zero value in 1 and 6 can be treated as the action signal onset times (on-time). Based on these times, segments of the EEG data can be selected from the “data” array by using given time offsets into time periods before and after the action signal on-time; thus, forming a fragment of EEG data associated with a participant’s given mental imagery. These “event data frames” should be associated with specific mental imageries by means of the “marker” values assumed immediately after the action signal’s on-switch. A dataset of these associated data frames can be used as the basis for subsequent analysis of the mental imagery in the included EEG data.

## Technical Validation

**Figure 3:**
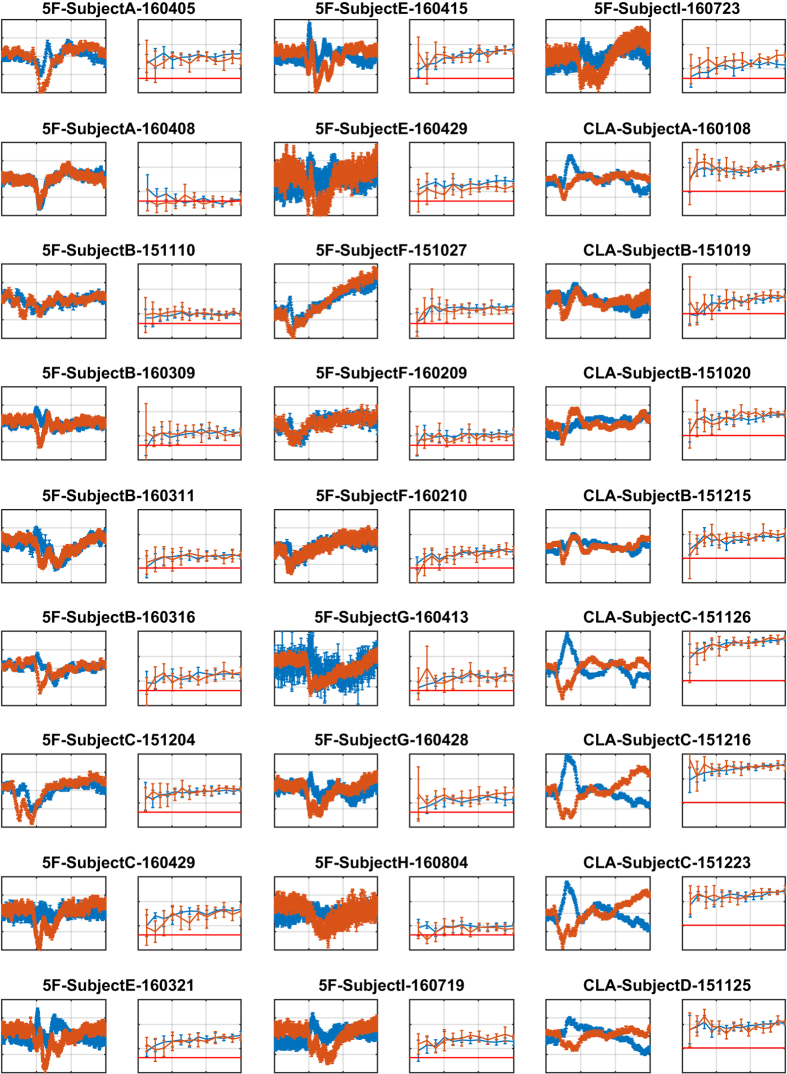
ERP curves of hand motor imagery responses on the C3 electrode, and imagery classification using the SVM-based classifier, for data files 5F-SubjectA-160405 through CLA-SubjectD-151125. The x-axis of all ERP figures is the action signal-locked time with the limits [0, 1500] ms relative to the action signal onset time, and the y-range is ±3 μV. The blue and the red curves are the right- and the left-hand motor imagery average ERPs, respectively. The classification plots show the standard learning curves of the all-class accuracy of the mental imagery classification vs. the number of samples used in training. The x-axis is the number of training samples ranging from 0 to 550 (100% of training set), and the y-axis is the classification accuracy with limits [0,1]. The blue and the red curves are the validation and the test dataset accuracies as a function of the number of training samples, and the straight red line is the chance level for each experiment.

**Figure 4:**
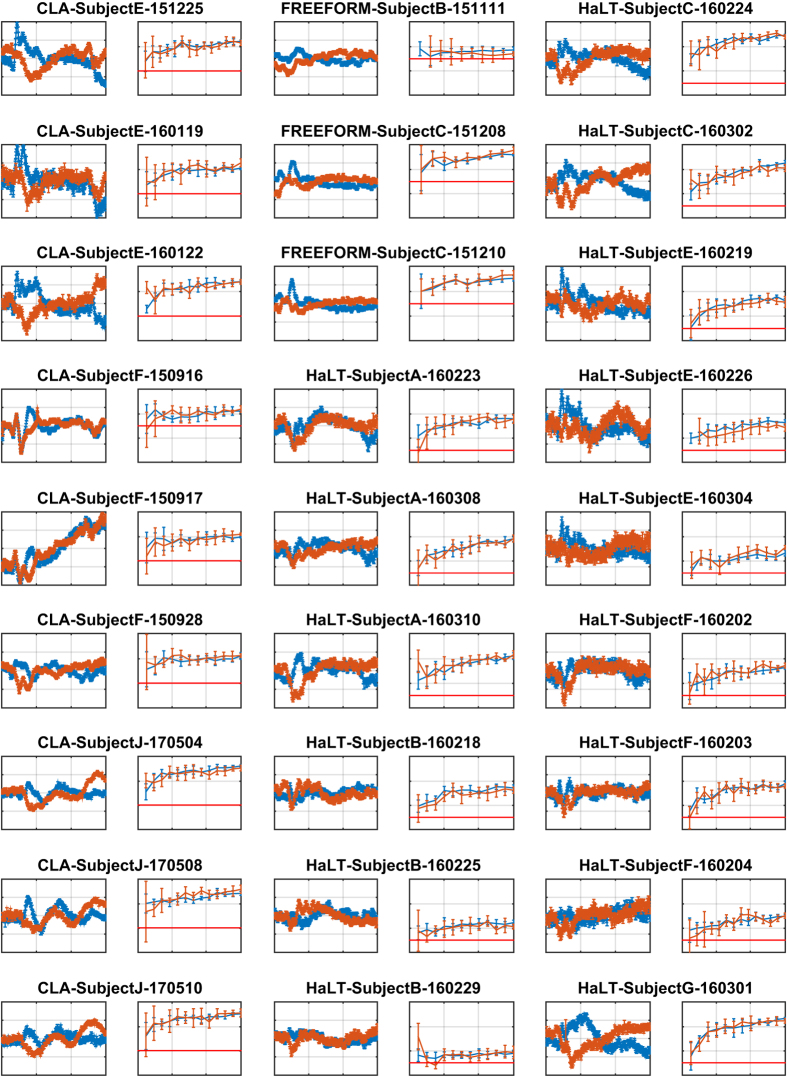
ERP curves corresponding to hand motor imagery responses on the C3 electrode, and classification using an SVM-based classifier for data files CLA-SubjectE-151225 through HaLT-SubjectG-160301. The axes are as described in Fig. 3.

**Figure 5:**
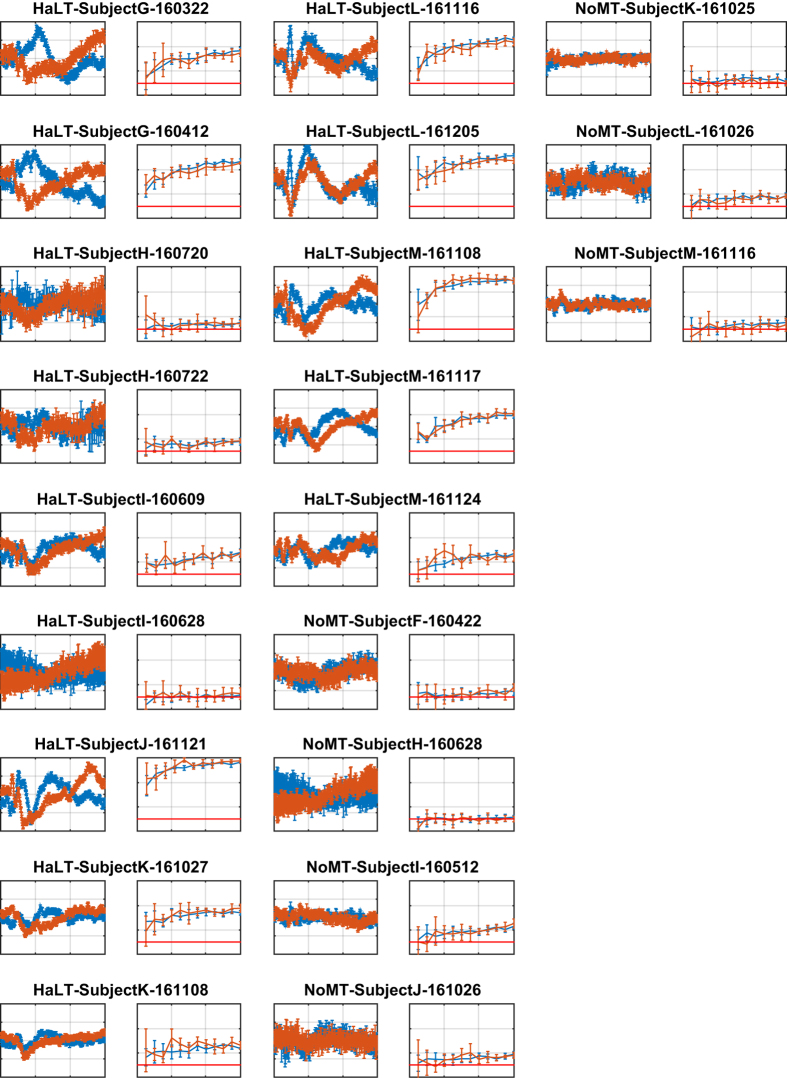
ERP curves of hand motor imagery responses on the C3 electrode, and classification using an SVM-based classifier for data files HaLT-SubjectG-160322 through NoMT-SubjectM-161116. The axes are as described in Fig. 3.

We performed a technical validation of the dataset by building and examining all stimulus-locked average event-related potential (ERP) curves for all different types of mental imageries, across all recording sessions, all input channels, and all mental imageries. ERP is a classical way of representing EEG responses^41–47^. We also carried out a classification of the mental imageries included in each data file using an SVM machine learning algorithm^48–53^ and an approach detailed in Mishchenko *et al.*^40^. The results of that validation are reported in Figs. 3, 4, 5.

We follow the methodology described in Mishchenko *et al.*^40^ for mental imagery classification study. Interested readers are requested to refer to that manuscript for details. Briefly, for each individual data file, the marker field of the included data record was processed to identify the onset times of all action stimuli as zero-to-non-zero change in the marker state. A fragment of the 21-channel EEG signal was then extracted from the raw EEG data in the record starting with the action stimulus onset time and continuing for 0.85 s immediately after (for 170 frames at a sampling rate of 200 fps). A 170-point discrete Fourier transform was then computed for each fragment to produce 86 complex Fourier transform amplitudes (FTA) for each EEG channel in each fragment, spanning the range at a granularity of 1.18 Hz. A low-pass 5 Hz filter was applied by keeping the five lowest amplitudes below 5 Hz (including 0 Hz). These were then converted into real representations by retaining the real and the imaginary part of each amplitude, with the exception of 0 Hz amplitude, which is always real, producing nine FTA features for each channel of each data fragment. These “Cartesian” FTA features for each event were combined from all EEG channels to generate a single vector of 189 features describing one mental imagery event. The dataset comprising all such mental imagery events was split randomly in 63-27-10% proportions for training, validation, and test sets. A standard voting-matrix multi-class SVM classifier was trained on the training set and evaluated on the validation (240 samples) and test (90 samples) datasets separately. This procedure (random dataset splitting, classifier training, and evaluation) was repeated five times for each data file to collect the confidence intervals of the cross-validated classification accuracies shown as the error bars in the learning curve plots in Figs 3, 4, 5.

Specifically, in Figs 3, 4, 5, rows 1, 3, and 5 show examples of the ERP curves for left- and right-hand MI for all recording sessions, observed over EEG channel C3. Motor imagery of left- and right-hand movements is among the most easily distinguishable in EEG BCI due to the contra-lateral nature of cortical regions responsible for the control of hand movements in the brain. We expected to see clear separation of these ERP curves in the majority of our recording session, as can indeed be observed in Figs 3, 4, 5 with exception of 5 F and NoMT recording sessions, where such separation is not expected by the nature of the recording sessions in those paradigms.

The motor imageries in 5 F are associated with the movements of the fingers on one hand, which are controlled essentially from the same area of the motor cortex and therefore may not separate very clearly in the ERP curves at channel C3. For 5 F recording sessions, we plotted the ERP curves of thumb vs. pinkie MI (Fig. 3) due to those having the largest difference. We expected to see some sort of response from the C3 cortex area in those plots, even if the ERP curves for different finger movements could not be clearly separated. Indeed, such a situation can be observed in Fig. 3.

In NoMT recording sessions, participants did not respond to presentations of action signals and merely passively watched the eGUI on the computer screen. We expected to see the ERP curves not differ significantly from noise, as is the case for those recording sessions.

**Figure 6:**
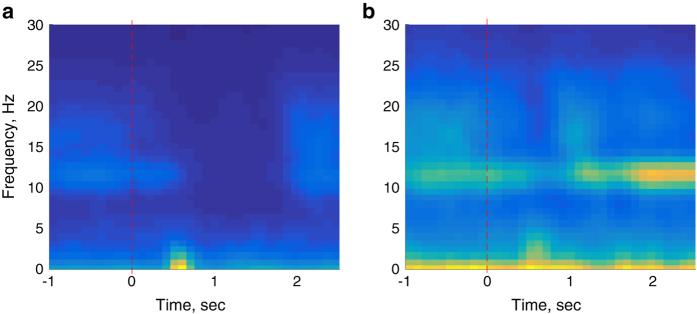
An example ERD/ERS calculation for left/right-hand motor and passive imageries for participant “Subject C” in the data record CLA-SubjetC-151126. (**a**) The spectral power distribution in the short-time Fourier transform of the EEG response in channel C3 for left-hand motor imagery of the participant. (**b**) The same for the passive imagery events. The red dashed line indicates the action stimulus onset time.

**Figure 7:**
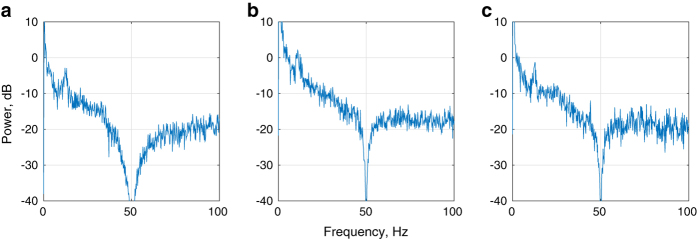
An example of EEG periodograms for different records in the dataset. (**a**) EEG periodogram for participant “Subject A”, input channel 6 (C4), from data record 5F-SubjectA-160408 (interaction paradigm 5 F). (**b**) EEG periodogram for participant “Subject A”, input channel 10 (O2), from data record CLA-SubjectA-160108 (interaction paradigm CLA). (**c**) EEG periodogram for participant “Subject C”, input channel 20 (Cz), from data record HaLT-SubjectC-160302 (interaction paradigm HaLT).

Note that although Figs 3, 4, 5 present only the left/right-hand ERPs for all data files, other types of analyses can be easily constructed from the raw data included in this dataset, such as focusing on ERD/ERS and mu-rhythms, as exemplified in Figs 6 and 7 shows examples of the spectral energy distribution in the EEG signal in different records for different EEG input channels and interaction paradigms in the dataset.

Rows 2, 4, and 6 in Figs 3, 4, 5 show the results of training the multi-class SVM classifier for the discrimination of different mental imagery states. The classification results are reported as learning curves, which describe the percentage of correctly classified events (y-axis, accuracy) within training, validation, and test datasets as a function of the number of mental imagery examples used for training the classifier (x-axis, training samples count). The random chance levels are indicated with solid red lines. We observe that the classification of the mental imageries can be performed well above the chance level for all data files (excluding NoMT).

**Figure 8:**
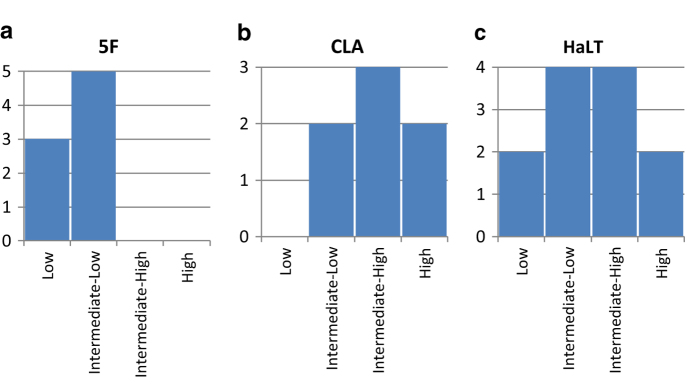
Participant distribution by observed BCI proficiency. (**a**) Participant BCI proficiency in the 5 F interaction paradigm. Discrimination of single finger motor imageries is difficult in EEG data, as demonstrated by the relatively low performance of the participants in this diagram. (**b**) Participant BCI proficiency in the CLA interaction paradigm. The discrimination of left- and right-hand motor and a passive imagery is relatively easy and all participants show performance substantially better than chance in this paradigm. (**c**) Participant BCI proficiency in the HaLT recording sessions. In this challenging BCI paradigm, we observed a wide accuracy distribution, from slightly above chance to almost 90%.

We quantified the variability in discrimination of mental imageries with respect to different interaction paradigms and different participants. EEG BCI performance variability in individual participants is well known in the literature and has been termed “BCI literacy”^46,54–56^. We observed that different participants demonstrated markedly different yet consistent EEG BCI performance in this dataset as well. The distribution of EEG BCI symbol discrimination accuracy across participants in 5 F, CLA, and HaLT interaction paradigms is summarized in Fig. 8. Participants were grouped into four categories of *low*, *intermediate-low*, *intermediate-high*, and *high* performing individuals. Those categories were defined by splitting the interval from the chance BCI classification level (i.e., 1/{Number of BCI states}) to 100% into four equal groups. Thus, for the 5 F paradigm, low performance was defined as anything better than 20% but worse than 40% accuracy of classification of the 5 finger MI. Intermediate-low performance was defined as 40 to 60% of such classification accuracy. Intermediate-high performance was defined as 60 to 80% classification accuracy, and high performance was defined as better than 80%. In the 5 F interaction paradigm, no participants managed to achieve performance better than intermediate-low, with three participants showing low performance and five participants achieving intermediate-low performance, for a total of eight participants (Fig. 8).

Similarly, for the CLA interaction paradigm, all participants demonstrated better than intermediate-low performance, defined here as better than 50% accuracy in discriminating left- and right-hand MI and the third passive state (random guess accuracy 33%). Specifically, two participants showed intermediate-low performance in separating those three states (between 50 and 67% accuracy), three participants demonstrated intermediate-high accuracy (67 to 83%), and two participants showed high performance (better than 83% accuracy), for a total of seven participants available in this interaction paradigm.

It should be noted that although the series of CLA recording sessions in the present dataset includes seven participants, HaLT recording sessions also contain the CLA mental imageries as a subset of their action stimuli. Thus, the performance of all participants in the CLA interaction paradigm can be assessed by including the corresponding parts of the HaLT recording sessions in the analysis. However, we did not pursue this here.

For the HaLT paradigm, we had a total of 12 participants with 29 recording sessions in the dataset. The HaLT panel in Fig. 8 shows the breakdown of the results of separating the six mental imageries in this paradigm by participant. Two participants showed low and high performance each, and four participants showed intermediate performance in each of the two groups, for a total of 12 participants.

We calculated the interaction paradigm-specific BCI performance measures for 5 F, CLA, and HaLT interaction paradigms. Values are expressed as mean classification accuracy of the included mental imageries averaged across all participants. Average accuracies were 43 ± 10% (mean ± SD) for the 5 F interaction paradigm, 75 ± 10% for the CLA paradigm, and 57 ± 20% for the HaLT paradigm (Table 6).

## Additional information

**How to cite this article**: Kaya, M. *et al.* A large electroencephalographic motor imagery dataset for electroencephalographic brain computer interfaces. *Sci. Data*. 5:180211 doi: 10.1038/sdata.2018.211 (2018).

**Publisher’s note**: Springer Nature remains neutral with regard to jurisdictional claims in published maps and institutional affiliations.

## Supplementary Material



## Figures and Tables

**Table 1:** Structure of the data record variable “o”.

**Data Record Variable**	
id	A unique alphanumeric identifier of the record
nS	Number of EEG data samples
sampFreq	Sampling frequency of the EEG data
marker	The eGUI [interaction record of the recording session]
data	The Raw [EEG data of the recording session]

**Table 2:** Abbreviations used in the data record naming convention.

**Abbreviation**	**Meaning**	**Explanation**
CLA	Classical (CLA) left/right hand MI	Paradigm#1
HaLT	Hand/leg/tongue (HaLT) MI	Paradigm#2
FREEFORM	Freestyle left/right hand MI	Paradigm#3
5 F	5 fingers (5 F) MI	Paradigm#4
NoMT	No imagery, visual stimuli only	Paradigm#5
SGLHand	Single hand	Recording sessions in 5 F paradigm performed for the motor imagery of fingers on one hand
HFreq	High frequency	Recording sessions recorded with 1000 Hz sampling rate setting in EEG 1200 as opposed to 200 Hz sampling rate used for all other recording sessions
Tong	Tongue	Identifies recording sessions including tongue MI
St	State	Indicates the total number of mental imageries present in the recording session
Inter	Interface	Indicates recording sessions performed using interactive user Interface, which differs from other recording sessions by a lower signal resolution (0.133μV vs. 0.01 μV) and dynamic range (121 μV vs. 2 mV)
LRHand	Left/right hand	Indicates recording sessions for MI of left and right hand movements
LRHandLeg Tongue	Left/right hand, leg and tongue	Indicates recording sessions focusing on MI of left and right hand, left and right leg, and tongue movements

**Table 3:** List of data files in the dataset grouped by recording session participant.

**Subject**	**Sex**	**Age group**	**Health condition**	**Prior BCI experience**	**BCI literacy level**	**Data**
A	M	20-25	Healthy	None	Intermediate- High	5F-SubjectA-160405
						5F-SubjectA-160408
						CLA-SubjectA-160108
						HaLT-SubjectA-160223
						HaLT-SubjectA-160308
						HaLT-SubjectA-160310
B	M	20-25	Healthy	None	Intermediate- Low	5F-SubjectB-151110
						5F-SubjectB-160309
						5F-SubjectB-160311
						5F-SubjectB-160316
						CLA-SubjectB-151019
						CLA-SubjectB-151020
						CLA-SubjectB-151215
						FREEFORM-SubjectB-151111
						HaLT-SubjectB-160218
						HaLT-SubjectB-160225
						HaLT-SubjectB-160229
C	M	25-30	Healthy	None	Intermediate-High	5F-SubjectC-151204
						5F-SubjectC-160429
						CLA-SubjectC-151126
						CLA-SubjectC-151216
						CLA-SubjectC-151223
						FREEFORM-SubjectC-151208
						FREEFORM-SubjectC-151210
						HaLT-SubjectC-160224
						HaLT-SubjectC-160302
D	M	25-30	Healthy	None	Intermediate- Low	CLA-SubjectD-151125
E	F	20-25	Healthy	None	Intermediate- Low	5F-SubjectE-160321
						5F-SubjectE-160415
						5F-SubjectE-160429
						CLA-SubjectE-151225
						CLA-SubjectE-160119
						CLA-SubjectE-160122
						HaLT-SubjectE-160219
						HaLT-SubjectE-160226
						HaLT-SubjectE-160304
F	M	30-35	Healthy	None	Intermediate- Low	5F-SubjectF-151027
						5F-SubjectF-160209
						5F-SubjectF-160210
						CLA-SubjectF-150916
						CLA-SubjectF-150917
						CLA-SubjectF-150928
						HaLT-SubjectF-160202
						HaLT-SubjectF-160203
						HaLT-SubjectF-160204
						NoMT-SubjectF-160422
G	M	30-35	Healthy	None	Intermediate-High	5F-SubjectG-160413
						5F-SubjectG-160428
						HaLT-SubjectG-160301
						HaLT-SubjectG-160322
						HaLT-SubjectG-160412
H	M	20-25	Healthy	None	Low	5F-SubjectH-160804
						HaLT-SubjectH-160720
						HaLT-SubjectH-160722
						NoMT-SubjectH-160628
I	F	25-30	Healthy	None	Low	5F-SubjectI-160719
						5F-SubjectI-160723
						HaLT-SubjectI-160609
						HaLT-SubjectI-160628
						NoMT-SubjectI-160512
J	F	20-25	Healthy	None	High	CLA-SubjectJ-170504
						CLA-SubjectJ-170508
						CLA-SubjectJ-170510
						HaLT-SubjectJ-161121
						NoMT-SubjectJ-161026
K	M	20-25	Healthy	None	Intermediate- Low	HaLT-SubjectK-161027
						HaLT-SubjectK-161108
						NoMT-SubjectK-161025
L	F	20-25	Healthy	None	High	HaLT-SubjectL-161116
						HaLT-SubjectL-161205
						NoMT-SubjectL-161026
M	F	20-25	Healthy	None	Intermediate- High	HaLT-SubjectM-161108
						HaLT-SubjectM-161117
						HaLT-SubjectM-161124
						NoMT-SubjectM-161116

**Table 4:** List of dataset files according to BCI interaction paradigm and mental imagery classification results from the SVM-based machine learning classifier.

**Paradigm**	**Data**	**Avg.**	**SD**
5 F	5F-SubjectA-160405	43%	10%
	5F-SubjectA-160408		
	5F-SubjectB-151110		
	5F-SubjectB-160309		
	5F-SubjectB-160311		
	5F-SubjectB-160316		
	5F-SubjectC-151204		
	5F-SubjectC-160429		
	5F-SubjectE-160321		
	5F-SubjectE-160415		
	5F-SubjectE-160429		
	5F-SubjectF-151027		
	5F-SubjectF-160209		
	5F-SubjectF-160210		
	5F-SubjectG-160413		
	5F-SubjectG-160428		
	5F-SubjectH-160804		
	5F-SubjectI-160719		
	5F-SubjectI-160723		
CLA	CLA-SubjectA-160108	75%	10%
	CLA-SubjectB-151019		
	CLA-SubjectB-151020		
	CLA-SubjectB-151215		
	CLA-SubjectC-151126		
	CLA-SubjectC-151216		
	CLA-SubjectC-151223		
	CLA-SubjectD-151125		
	CLA-SubjectE-151225		
	CLA-SubjectE-160119		
	CLA-SubjectE-160122		
	CLA-SubjectF-150916		
	CLA-SubjectF-150917		
	CLA-SubjectF-150928		
	CLA-SubjectJ-170504		
	CLA-SubjectJ-170508		
	CLA-SubjectJ-170510		
FREEFORM	FREEFORM-SubjectB-151111		
	FREEFORM-SubjectC-151208		
	FREEFORM-SubjectC-151210		
HaLT	HaLT-SubjectA-160223	57%	20%
	HaLT-SubjectA-160308		
	HaLT-SubjectA-160310		
	HaLT-SubjectB-160218		
	HaLT-SubjectB-160225		
	HaLT-SubjectB-160229		
	HaLT-SubjectC-160224		
	HaLT-SubjectC-160302		
	HaLT-SubjectE-160219		
	HaLT-SubjectE-160226		
	HaLT-SubjectE-160304		
	HaLT-SubjectF-160202		
	HaLT-SubjectF-160203		
	HaLT-SubjectF-160204		
	HaLT-SubjectG-160301		
	HaLT-SubjectG-160322		
	HaLT-SubjectG-160412		
	HaLT-SubjectH-160720		
	HaLT-SubjectH-160722		
	HaLT-SubjectI-160609		
	HaLT-SubjectI-160628		
	HaLT-SubjectJ-161121		
	HaLT-SubjectK-161027		
	HaLT-SubjectK-161108		
	HaLT-SubjectL-161116		
	HaLT-SubjectL-161205		
	HaLT-SubjectM-161108		
	HaLT-SubjectM-161117		
	HaLT-SubjectM-161124		
NoMT	NoMT-SubjectF-160422		
	NoMT-SubjectH-160628		
	NoMT-SubjectI-160512		
	NoMT-SubjectJ-161026		
	NoMT-SubjectK-161025		
	NoMT-SubjectL-161026		
	NoMT-SubjectM-161116		

**Table 5:** Explanation of the numerical codes used in recording session interaction records.

**CLA, HaLT, FreeForm, NoMT**		**5 F**	
**“marker” code**	**meaning**	**“marker” code**	**Meaning**
1	Left hand	1	Thumb
2	Right hand	2	Index finger
3	Passive/neutral	3	Middle finger
4	Left leg	4	Ring finger
5	Tongue	5	Pinkie finger
6	Right leg		
91	Session break		
92	Experiment end		
99	Initial relaxation		

**Table 6:** Mean mental imagery classification accuracy in the different BCI interaction paradigms.

**Interaction paradigm**	**All-subject mean classification accuracy**	**All-subject standard deviation**	**Highest chance accuracy *p = *0.95**	**Subjects**	**Recording sessions**	**Interaction segments**
CLA	75%	10%	38.8%	7	17	51
5 F	43%	10%	24.6%	8	19	57
HaLT	57%	20%	21.0%	12	29	87
